# Supplementary material for: A UMLS-based spell checker for natural language processing in vaccine safety
Source: BMC Med Inform Decis Mak. 2007 Feb 12;7:3. doi: 10.1186/1472-6947-7-3 (PMC1805499; doi:10.1186/1472-6947-7-3)
Supplement: Additional file 13 — RAP application directory. Sets up directory for RAP (RDF application for PHP) source code [file 1472-6947-7-3-S13.gz › rap/doc/tutorial/introductionToRAP.htm]

Introduction to RAP


### RDF API for PHP V0.9.1

# Introduction to RAP

This document is part of the RAP - Rdf API for PHP documentation.

Daniel Westphal, Chris Bizer  
October 2004

## Table of Contents

1. Overview
2. The MemModel
3. The DbModel
4. The Infmodels (InfModelF,
   InfModelB)
5. The ResModel
6. The OntModel

## Overview

RAP - RDF API for PHP is a Semantic Web toolkit for PHP developers. RAP started
as an open source project at the Freie Universität Berlin in 2002 and has
been extended with internal and external code contributions since then. Its
latest release includes:

- a statement-centric API for manipulating RDF graphs as a set of statements
- a resource-centric API for manipulating RDF graphs as a set of resources
- integrated RDF/XML, N3 and N-TRIPLE parsers
- integrated RDF/XML, N3 and N-TRIPLE serializers
- in-memory or database model storage
- support for the RDQL query language
- an inference engine supporting RDF-Schema reasoning and some OWL entailments
- an RDF server providing similar functionality as the Joseki
  RDF server
- a graphical user-interface for managing database-backed RDF models
- support for common vocabularies

RAP can be used under the terms of the GNU
LESSER GENERAL PUBLIC LICENSE (LGPL)   
and can be downloaded from http://sourceforge.net/projects/rdfapi-php/

RAP offers two different programming interfaces for manipulating RDF graphs:
The statement-centric **Model API** which allows you to manipulate
an RDF graph as a set of statements; and the resource-centric **ResModel
API** for manipulating an RDF graph as a set of resources.

The Model API supports adding, deleting, and replacing statements inside a
model as well as adding entire models. StatementIterators allow sequential access
to all statements within a model. There are four different implementations of
the Model API:

| Model | Functionality |
| --- | --- |
| MemModel | Model storing its RDF graph in memory. MemModel is fast, but doesn't support inference. |
| DbModel | Model storing its RDF graph in a relational database. No inference support. |
| InfModelF | Forward-chaining inference model storing its base graph and inferred triples in memory. |
| InfModelB | Backward-chaining inference model storing its base graph in memory and creating inferred triples on the fly. |

The resource-centric ResModel API represents RDF graphs as resources having
properties. The ResModel API is very similar to the Jena
Model API allowing programmers, which are used to Jena, to easily write
RAP code. The ResModel API is implemented on top of the Model API. ResModels
are always having a underlying MemModel, DbModel, InfModelF or InfModelB and
are only providing a resource-centric view on this model. There are two implementations
of the ResModel API:

| Model | Functionality |
| --- | --- |
| ResModel | Basic implementation of the ResModel API |
| OntModel | OntModel provides a ResModel implementation extended with RDF-Schema specific methods like hasSuperProperty(), addDomain() and listInstances(). |

The following sections are explaining the different RAP models in more detail.
Further tutorial and RAP usage examples are found on the RAP Documentation overview page.

## The MemModel

The MemModel implementation stores statements in an array in the system memory.
If a new statement is added to the model, it's appended to the array and added
to a statement index in order to accelerate queries. MemModel is the fastest
of all Model API implementations.

Further information about using the MemModel can be found in the "Using
RAP's Statement-Centric MemModel API" tutorial and in the MemModel
PHPDoc.

## The DbModel

The DbModel implementation stores statements in an relational database. DbModel
supports a wide range of different databases by using the ADODB database abstraction
layer. The core of RAP's database backend are the classes DbStore and DbModel.
The former represents all models stored in a database, whereas the latter provides
methods for manipulating these models.

RAP uses a denormalized database schema, where all statements are written into
a single statement table. We compared the performance of this solution with
a normalized layout. Running benchmarks has shown that the denormalized schema
was 2-3 times faster than the normalized one. The trade-off between better performance
and increased database size was acceptable for medium-sized RDF models.

Further information about using the DbModel can be found in the tutorial about
"Database Persistence" and
in the DbModel PHPDoc.

## The InfModels

InfModel extends MemModel by adding the ability to infer additional statements.
Up to our knowledge, InfModel is the only reasoning engine implemented in PHP.
The performance of InfModel can't compete with the performance of reasoning
engines implemented in Java or C, because PHP still isn't a very fast language.
But for medium sized models and relatively small RDF-S schemata, InfModel works
fine. InfModel supports the following RDF-S and OWL constructs:

- rdfs:subclass
- rdfs:subproperty
- rdfs:range
- rdfs:domain
- owl:sameAs
- owl:inverseOf

Additionally, the following inference rules from the W3C
RDF Semantics Recommendation are supported:

- RDFS Rule 6
- RDFS Rule 8
- RDFS Rule 10
- RDFS Rule 12
- RDFS Rule 13

You can activate and deactivate the different rules separately in constants.php.
Turning off rules that you don't need improves the performance of InfModel.

The abstract class InfModel is extended by two subclasses:

- InfModelF, which uses a forward chaining inference algorithm.
- InfModelB, which uses a backward chaining inference algorithm.

The pros and cons of both algorithms are described below:

### The InfModelF

InfModelF uses a forward chaining inference algorithm: If a new statement from
the RDFS or OWL namespace is added to the model, a corresponding InfRule is
added to the model's rule-base. The rule has a trigger and an entailment. If
a new statement, which matches the trigger of an InfRule, is added to the model
then the entailment will be recursively computed until no more rule-triggers
match the statement or statements inferred from it. The base statement and all
inferred statements are added to the model.

Materializing all possible inferred statements means that adding base statements
to the model is relatively slow. On the other hand find operations are very
fast, because the find method just has to look into the statement index and
return the matching statements (including inferred statements).

Thus you should use InfModelF

- if your model doesn't change much and
- you are going to query the model a lot.

Further information about using the InfModelF can be found in the InfModelF PHPDoc.

### The InfModelB

InfModelB uses a backward chaining inference algorithm, meaning that no inferences
are done when a new statement is added to the model. When a query is executed
against the model later, only the necessary inferences for this query are done:
A find-patter is executed with the supplied parameters first. Afterwards it
is checked against an inference-rules index, if there are any rules that could
produce statements that would match the find-pattern. If there are such rules,
the find-pattern is rewritten an a new search is done. The new statements are
inferred and added to the result. This iterative process will be repeated until
there are no rules left that could produce matching statements.

InfModelB recognizes subclassing loops in ontologies. It also uses some shortcuts
which speed up the search process: It recognizes branches in the ontology that
don't lead to any additional statements and avoids running into these 'dead
ends' a second time.

You should use InfModelB:

- if your model changes a lot,
- if you don't want to materialize all inferred statements,
- if you are having a lot of RDF-S statements or
- if you are not going to execute too many queries against the model.

Further information about using the InfModelB can be found in the InfModelB PHPDoc.

## The ResModel

ResModel provides a resource-centric view on an underlying Model. ResModels
represent an RDF graphs as a set of resources having properties, similar to
the Jena Model API.
The ResModel API is implemented on top of the Model API. Thus ResModels are
always having a underlying MemModel, DbModel, InfModelF or InfModelB and are
only providing a resource-centric view on this model.

Each ResModel method call is translated into a find()-, add()-, or remove()-call
to the underlying model. Thus using ResModel is slightly slower than using the
underlying model directly. The ResModel API also provides support for special
resources like rdf:containers and rdf:collections. There is no caching done
between the layers.

Further information about using the ResModel can be found in the tutorial about
"Using RAP's Resource-Centric ResModel
API" and in the ResModel PHPDoc.

## The OntModel

OntModel API provides a ResModel implementation extended with RDF-Schema specific
methods like addSubClass(), listSubClasses(), hasSuperProperty(), addDomain()
and listInstances().

OntModel just provides a more convenient API for working with RDF-S models,
but doesn't do any inference by itself. If you also need RDF-S inference
you have to combine OntModel with an underlying InfModelF or InfModelB. If you
don't need inference you can combine OntModel with an underlying MemModel
or DbModel.

Further information about OntModel can be found in the tutorial about "Using
RAP's Ontology-Centric OntModel API" and in the OntModel PHPDoc.
